# Autobiographical Memory Increases Pupil Dilation

**DOI:** 10.1515/tnsci-2019-0044

**Published:** 2019-12-19

**Authors:** Mohamad El Haj, Steve M. J. Janssen, Karim Gallouj, Quentin Lenoble

**Affiliations:** 1 Nantes Université, Univ Angers, Laboratoire de Psychologie des Pays de la Loire (LPPL - EA 4638), F-44000 Nantes, France; 2 Unité de Gériatrie, Centre Hospitalier de Tourcoing, Tourcoing, France; 3 Institut Universitaire de France, Paris, France; 4 The University of Nottingham–Malaysia Campus, Semenyih, Malaysia; 5 Univ. Lille, CNRS, CHU Lille, UMR 9193 - SCALab - Sciences Cognitives et Sciences Affectives, F-59000 Lille, France

**Keywords:** autobiographical memory, pupil, pupil dilation, pupillometry

## Abstract

**Background:**

Pupil activity has been widely considered as a “summed index” of physiological activities during cognitive processing.

**Methodology:**

We investigated pupil dilation during retrieval of autobiographical memory and compared pupil diameter with a control condition in which participants had to count aloud. We also measured pupil diameters retrieval of free (i.e., first memory that comes to mind), positive, and negative memories (memories associated, respectively, with the words “happy” and “sad”).

**Results:**

Analyses demonstrated larger pupil diameters during the free, positive, and negative autobiographical memory retrieval than during the control task. Analyses also demonstrated no significant differences in pupil diameters across the three autobiographical memory conditions.

**Conclusion:**

These outcomes demonstrate that, compared with counting, autobiographical retrieval results in a larger pupil size. However, the emotional valence of memories yields non-significant effect on pupil diameters. Our findings demonstrate how autobiographical memory retrieval yields pupil dilation.

## Autobiographical memory increases pupil dilation

This paper investigates whether retrieval of autobiographical memory (i.e., memory regarding personal information) increases pupil dilation. Given this objective, we briefly the describe physiological bases of pupil dilation. We then describe research demonstrating how memory can modulate pupil dilation. As we will highlight, there is a large body of research demonstrating how memory can shape pupil dilation, but there is a lack of studies on pupil dilation during the retrieval of autobiographical memory.

To begin with the physiological basis of pupil dilation, the pupil is the opening area of the iris that allows light to enter the eye and reach the retina. The pupil is controlled by two sets of smooth muscles in the iris, namely the sphincter muscles and dilator muscles [1, 2]. Whereas the sphincter muscles decrease the diameter of pupil, the dilator muscles increase it. These muscles serve to optimize vision by modulating the amount of light that reaches the retina; whereas the pupil dilates in darker conditions, it constricts in brighter conditions. Pupil diameter typically varies from 1.5 to nine mm; pupil reacts to stimulation in about 200 ms and, in standard light conditions, pupil diameter is about three mm [3].

Pupil dilation is mediated by simultaneous activation of the sympathetic system and inhibition of the parasympathetic system, more precisely, cortical inhibition of the parasympathetic oculomotor nucleus [4]. Pupil dilation has been shown to be mediated by activity of the locus coeruleus–noradrenaline system [5], which plays an important role in cognitive processes [6]. More precisely, pupil dilation is mediated by activation of neurons in the locus coeruleus which supply noradrenaline to the eyes and brain; in the eye, the noradrenaline regulates pupil dilation, and, in the brain, it regulates attention [7, 8]. Pupil dilation is not solely mediated by neurophysiological processes but also by cognitive and emotional processes.

In a seminal work, Hess and Polt [9] reported that pupil was larger in response to positive images and smaller in response to negative images. Subsequent research has demonstrated that pupil typically dilates when participants are in conditions of increased emotion [10, 11] or attention [12]. This dilation has been attributed to the cognitive load of the task [13]. The effect of cognitive load on pupil dilation has been also observed on memory tests. Kahneman and Beatty [13] reported increased pupil dilation in response to the increased difficulty of a working memory task. Similar findings were reported by subsequent studies using the span tasks, on which participants are typically invited to repeat a string of numbers. The studies reported that pupil dilation increases with each digit retained in digit span tasks until the length of the digits exceeds the capacity of working memory, at which pupil diameter begins to plateau or even diminish [14, 15, 16, 17, 18].

Pupil dilation has been also studied in recognition memory [19, 20]. In recognition memory, participants typically make old/ new judgments on previously studied and new information. Using these procedures, Gardner, Philp [21] reported increased pupil dilation when participants processed old information during recognition memory tasks. They suggested that pupil dilation mirrors mental effort related to encoding and retrieval of information from memory rather than the general level of mental effort, as proposed by the cognitive load theory of Kahneman and Beatty [13]. In a similar vein, Vo, Jacobs [22] attributed pupil dilation in recognition memory tasks to the cognitive demands of recognizing old information compared with rejecting new information. According to Vo, Jacobs [22], recognition of old information requires retrieval of qualitative contextual information about the encoding episode (e.g., when and where the information was encoded), whereas correct rejection does not require this effortful retrieval. Also, Otero, Weekes [23] suggested that pupil dilation for old information depends on the strength of memory traces upon which recognition judgment is made. These suggestions can explain why, in recognition memory, pupil dilates more for information judged as old versus information judged as new [20, 24, 25, 26].

The above-mentioned research suggests that, on recognition memory tasks, pupil dilates more when participants process old stimuli compared with new stimuli. Inspired by this research, studies consider pupil dilation as an indicator of encoding and retrieval of long-term memory as pupil dilation predicts the strength of subsequent memory [27] and discriminating between familiar and recollected information [28]. The latter discrimination was evaluated by Kafkas and Montaldi [28] who reported a linear effect in which pupil dilation increased linearly from new to familiar and recollected information, with recollection producing the highest levels of pupil dilation and novelty the lowest, with familiarity falling somewhere in between.

Pupil dilation, as observed during recognition memory tasks, can be modulated by expectations; an assumption proposed by Mill, O’Connor [29] who reported increased pupil dilation during recognition of unexpected information compared to expected information. The authors suggested that dilation responses during memory recognition are mediated by expectations; more precisely, they suggested that expected information yields an acontextual sense of recollection whereas unexpected information yields a recollection of contextual information, and consequently, pupil dilation. This suggestion can be supported by the dual processing model according to which recollection-based decisions, compared to familiarity-based memory decisions, yields retrieval of contextual information about the encoding episode [30].

Together, there is a substantial body of research on the effects of memory on pupil dilation. This research reported the increased pupil dilation in response to increased difficulty of memory processing [13, 14, 15, 16, 17, 18]. This research also reported that, on recognition memory tasks, pupil dilates more when participants process old stimuli compared to new stimuli [20, 24, 25, 26].

## The Present Study

Although the previous research is useful in understanding the effects of memory processing on pupil dilation, there is, to the very best of our knowledge, a lack of research on pupil dilation during autobiographical memory retrieval. This issue is important because the study of autobiographical memory is concerned with how people remember personal events. Autobiographical memory allows the recall of events that are relevant to one’s identity and sense of self [31, 32].

To this aim, we compared pupil dilation during autobiographical memory retrieval and during a control task in which participants had to count aloud. We also investigated pupil dilation following the emotional valence of memories for two reasons. First, autobiographical memory has been intimately associated with emotion [31]. Second, pupil dilation has been found to be sensitive to emotion [9, 10, 11]. Therefore, pupil dilation can be influenced by the emotional tone of memory. As for hypotheses, we expected a larger pupil diameter during autobiographical memory retrieval than during the control task. We also expected the large pupil diameter during retrieval of emotional memories than during the retrieval of free memories.

## Method

### Participants

The study included 36 graduate/undergraduate students from the University of Nantes (19 females, *M* age = 24.21 years, *SD* = 6.14, *M* education = 14.61 years, *SD* = 4.74). Participants were native French speakers. Among the original sample (*N* = 44), pupil data of two participants were corrupted, three participants were excluded owing to signal loss during recording, and three participants were excluded owing to previous psychiatric or neurological disorders. This final sample size was determined a priori using G*Power [33]. The calculation was conducted for repeated measures (four within-subjects measurements) ANOVA tests, based on 95% power, an estimated probability of making Type I error as .05, and a medium effect size of 0.25 [34]. In the final sample size, no significant differences were observed regarding gender [*X*^2^ (1, *N* = 36) = .87, *p* = .50].

**Informed consent**: Informed consent has been obtained from all individuals included in this study.

**Ethical approval**: The research related to human use has been complied with all the relevant national regulations, institutional policies and in accordance the tenets of the Helsinki Declaration, and has been approved by the authors’ institutional review board or equivalent committee.

## Procedures and Materials

**Figure 1:**
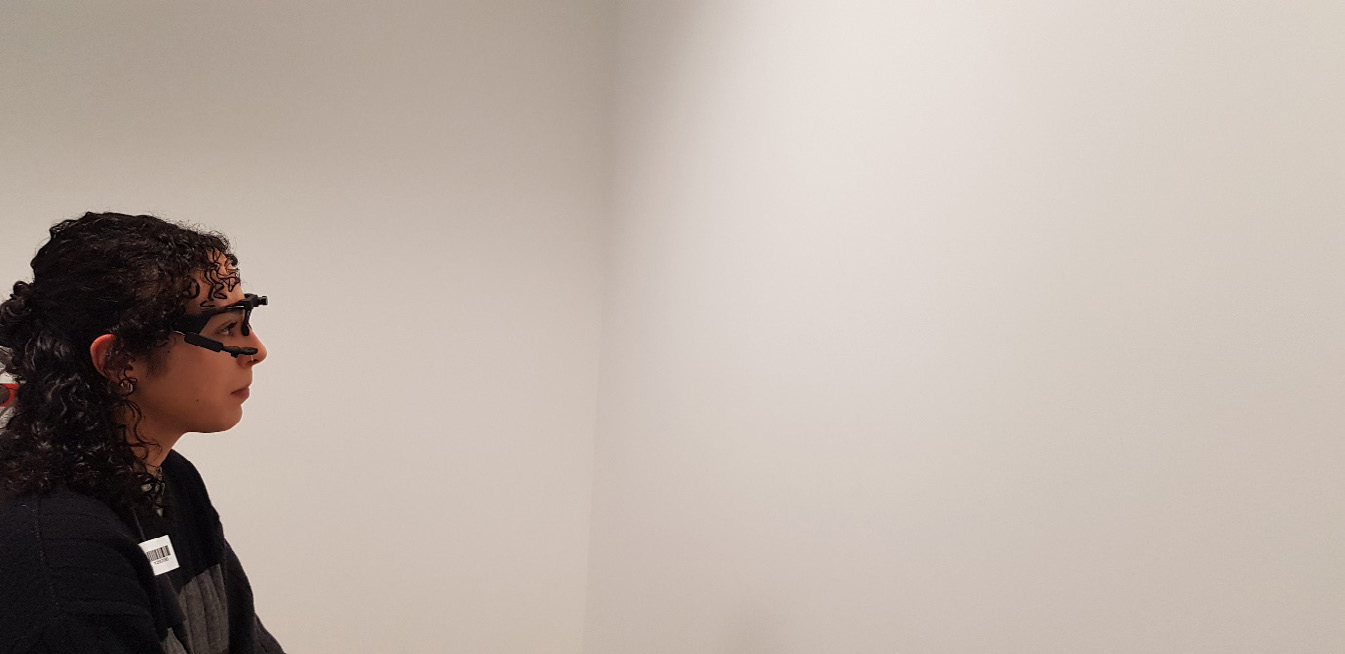
During the four conditions (i.e., free autobiographical recall, positive autobiographical recall, and negative autobiographical recall, as well as counting as a control condition), participants wore eye-tracking glasses and faced a white wall

Generally speaking, procedures consisted of four conditions (i.e., free autobiographical recall, positive autobiographical recall, and negative autobiographical recall, as well as counting as a control condition). During these conditions, the participants wore eye-tracking glasses and faced a white wall (see Figure 1). Participants were tested individually and were informed that the experiment was concerned with memory. However, in order not to influence their performance, the participants were not provided with further details about autobiographical memory or pupil dilation.

In the autobiographical conditions, participants were invited to verbally generate three autobiographical events. Prior to each autobiographical event, participants were instructed to remember in detail an event related to the cue. They were also instructed that the event had to be personally experienced in the past and that the description had to be precise and specific (e.g., when and where the event occurred, what they were doing during it, who was present, what their feelings were). The participants were invited to retrieve one free memory, one positive memory, and one negative memory. For free memory, they were instructed to verbally describe the first memory that comes to mind. For the positive memory, they were instructed to verbally describe a memory associated with the word “happy”. For the negative memory, they were instructed to verbally describe a memory associated with the word “sad”. One minute was allocated to generate each autobiographical memory, and the duration was made clear beforehand so that participants could structure their memories accordingly.

On the control condition, participants were invited to count aloud to count from 1 “in their own time” until the experimenter said “Stop”. The latter signal was provided after one minute of counting. This control condition was chosen, because, like autobiographical memory, it relies on verbal behaviour, therefore, any potential differences in pupil dilation between the two conditions would not be the result of verbal behaviour.

Autobiographical memory retrieval and counting occurred while pupil dilation was recorded. Recording was stopped directly after memory retrieval and counting. Participants wore eye-tracking glasses. These glasses (Pupil Lab) are a remote pupil-tracking system that uses infrared illumination with a gaze position accuracy of < 0.1° and a 200 Hz sampling rate. Recording was processed with the Pupil Capture software. Prior to each condition (i.e., free, positive, and negative memories, as well as for counting) calibration was made by inviting participants to fixate on a black cross (a 5 x 5 cm cross, printed on an A4 white paper fixated at the wall center) and the cross was defined as a calibration reference, needless to say, that the cross was withdrawn after calibration. The experiment occurred in a quiet room at the psychology department of the University of Nantes. Blinds were closed and the lightness of the room (60-watt fluorescent lamp) was the same in the two conditions to ensure that differences in pupil dilation were not caused by differences in retinal illumination. Participants were seated in front of a white wall and the distance between the subjects and wall was approximately 30 to 50 cm. Participants were invited not to look outside the wall but were free to explore all parts of it. The wall displayed no visual stimuli (e.g., drawings, windows). We defined pupil dilation as the average dilation during each trial; this interval was chosen to encompass the full retrieval of memories, as well as the full counting in the control condition.

## Results

We compared pupil dilation across counting and the three autobiographical trials (i.e., the “free”, “positive”, and “negative” memories) with repeated measures ANOVA, followed up by *t*-tests pair-wise comparisons. For *t*-tests, we provided effect sizes by using Cohen’s *d* [34]: 0.20 = small, 0.50 = medium, 0.80 = large. For all tests, the level of significance was set as *p* ≤ 0.05.

**Figure 2:**
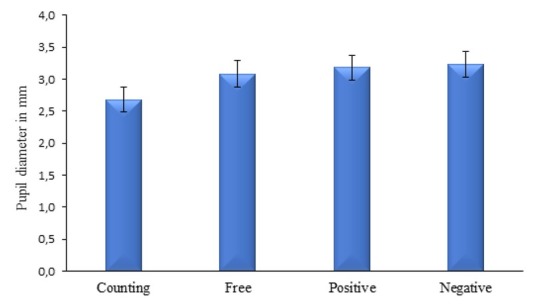
Means of pupil diameters during counting and during retrieval of “free” (i.e., first memory that comes to mind), and “positive” and “negative” memories (memories associated, respectively, with the words “happy” and “sad”). Error bars are 95% within-subjects confidence intervals.

The pupil diameter data is provided in Figure 2. Analysis showed significant differences between pupil diameters across the four trials (i.e., counting, “free”, “positive”, and “negative” memories), *F*(3, 105) = 6.14, *p* = .001, η^2^ = .15. This effect was solely caused by the difference between the counting and the three autobiographical retrieval conditions. Wilcoxon tests showed larger pupil diameter during free autobiographical retrieval than during counting [*t*(35) = 2.76, *p* = .009, Cohen’s *d* = .56], larger pupil diameter during positive autobiographical retrieval than during counting [*t*(35) = 4.31, *p* < .001, Cohen’s *d* = .66], and larger pupil diameter during negative autobiographical retrieval than during counting [*t*(35) = 3.52, *p* = .001, Cohen’s *d* = .59]. However, no significant differences in pupil diameter were observed between free and positive autobiographical retrieval [*t*(35) = .76, *p* > .10, Cohen’s *d* = .17], between free and negative autobiographical retrieval [*t*(35) = .89, *p* > .10, Cohen’s *d* = .18], and between positive and negative autobiographical retrieval ([*t*(35) = .35, *p* > .10, Cohen’s *d* = .09].

## Discussion

We compared pupil diameter during the retrieval of three autobiographical memories and a control task, in which participants had to count. Analyses demonstrated larger pupil diameters during free, positive, and negative autobiographical memory retrieval than during the control task. Analyses also demonstrated no significant differences in pupil diameter across the three autobiographical memory conditions (i.e., free, positive, and negative). These outcomes demonstrate that, compared to counting, autobiographical memory retrieval results in larger pupil size. However, the emotional valence of the recalled memories yields no significant effect on pupil diameter.

The main finding is the larger pupil diameter during autobiographical memory retrieval. This study is the first one to demonstrate that the retrieval of personal information yields dilation of the pupils. More specifically, it demonstrates that pupil dilation, as observed in the literature on the memory of general information (e.g., memory for words), can also be extended to self-related information (i.e., autobiographical memory).

## Effects of Memory on Pupil Dilation

To begin with the contribution of our study to the literature on the effects of memory on pupil dilation (and as mentioned in the introduction), there is a large body of research reporting increased pupil dilation in response to increased difficulty of memory processing [13, 14, 15, 16, 17, 18]. This research also reported that, on recognition memory tasks, pupil dilates more when participants process old stimuli compared to new stimuli [20, 24, 25, 26]. This body of literature is, however, mainly concerned by recognition memory and working memory.

Research on pupil dilation and working memory has been mainly interested in how updating in working memory yields pupillary responses. In this research, updating has been defined as the ability to integrate incoming information with information that is currently held in working memory [35]. Using span tasks, research has shown increased pupil dilation with the increased number of to-be-tracked information [17, 18, 36]. Similar findings are reported by research using the n-back task. This working memory task requires participants to indicate whether the currently presented information is the same as information presented n trials back [35]. Using this task, studies have demonstrated that pupil dilation increases with increasing n [37, 38, 39]. This line of research has attributed the pupil dilation to the cognitive load of the tasks.

Interestingly, Hogervorst, Brouwer, and van Erp (2014) have shown that pupil dilation can be sufficient to distinguish high cognitive load from the low cognitive load. When compared to other physiological measures, such as electroencephalography, respiration, skin conductance, or cardiac rhythms, pupil dilation discriminates high and low cognitive load with 80% accuracy [40]. These findings demonstrate that pupil dilation is a reliable index of cognitive load associated with memory.

Pupil dilation, as observed in our study, may be attributed to the cognitive load associated with autobiographical memory retrieval. This attribution is supported by the above-mentioned research, suggesting how pupil dilation mirrors the cognitive load of working memory tasks [40]. Our attribution is also supported by the Self Memory System [31], according to which autobiographical memory retrieval requires a controlled reconstruction. This controlled reconstruction is required to retrieve not only the targeted information but also the context in which this information was encoded [30, 41, 42, 43]. In our view, pupil dilation during autobiographical memory, compared to counting, may be attributed to the general cognitive load of memory retrieval and, more specifically, to the cognitive effort required to construct the context in which the information was previously encoded [22, 29]. Alternatively, verbalizations were perhaps more complex in the autobiographical memory conditions than in the counting condition and this complexity might, therefore, rather than the autobiographical memory retrieval, drove the pupil dilation.

To summarize, there is a large body of literature on pupil dilation during memory a recognition and working memory tasks. Our findings contribute to this literature by demonstrating how the retrieval of self-related information (i.e., autobiographical memory) yields pupil activation as well. This activation may be attributed to the cognitive effort as required to reconstruct the memories and, more specifically, to retrieve the contextual information about the encoding episode.

## Pupil Dilation as Physiological Measure

Our findings demonstrate how pupil dilation can be used as a physiological evaluation of this retrieval. Physiological correlates of autobiographical memory have been mainly evaluated with regard to brain activity. In this research, autobiographical memory has been associated with a “core network” of brain areas, including the hippocampus, medial and ventrolateral prefrontal cortex, posterior cingulate, and temporoparietal junction [44, 45].

Unlike the large body of research on brain activity during autobiographical memory retrieval, little research has attempted to evaluate cardiovascular or electrodermal activities during this retrieval. The latter research has demonstrated significant variations in cardiovascular and electrodermal activity during autobiographical memory retrieval [46]. In a similar vein, Robertson, Swickert [47] reported variations in blood pressure during autobiographical memory retrieval. Considering aging, Labouvie-Vief, Lumley [48] reported a lower heart rate in older adults than in younger participants during autobiographical memory retrieval.

Another physiological evaluation of autobiographical memory is facial expressions. Research has demonstrated variations of facial expressions during autobiographical memory retrieval [49, 50, 51]. This research has suggested that emotional facial expressions may reflect the physiological states that were experienced in the encoded events [52].

Autobiographical memory has not been only associated with the brain, cardiovascular, electrodermal, and facial expressions activities, but also with eye movements [53]. Eye movements during autobiographical memory retrieval were evaluated in a study in which participants had to retrieve autobiographical memories and, as a control condition, count aloud [54]. Results demonstrated a lower number of fixations but a higher number, larger amplitude, and longer duration of saccades in the autobiographical condition than in the control condition. Eye movement activity was attributed to the attempt of the visual system to create and manipulate mental representations of the memories [54]. A similar suggestion was made in a study comparing eye movements during retrieval of neutral memories and emotional memories [55]. Another study reported that autobiographical memory retrieval and future episodic thinking were accompanied by similar durations of fixations and saccades, as well as similar amplitudes of saccades [56].

Together, physiological correlates of autobiographical memory have been evaluated with regard to facial expressions and brain, cardiovascular, electrodermal, and eye movement activity. Our study contributes to this literature by demonstrating that autobiographical memory can also be evaluated with pupil dilation.

## Effects of Emotion on Pupil Dilation

Although we have found that pupil diameter was larger during autobiographical memory retrieval than during the control task, our findings demonstrated no significant differences between pupil dilations across the retrieval of freely recalled, positive, and negative memories. This finding seems to contradict a body of research demonstrating how pupil dilation is dependent on emotion. Research has demonstrated that pupils tend to dilate when participants view positive or negative images relative to neutral images [10]. This dilation has been replicated in many studies in response to emotional images [57, 58, 59], emotional video-clips [60], as well as facial expressions [61, 62].

To resolve the apparent contradiction between our findings and the previous literature on the pupil responsivity to emotion, it would be of interest to highlight differences between our study and the studies in the literature. Our study is concerned with autobiographical memory, a memory system that has been intimately associated with emotion [31]. Except psychopathological conditions [63], autobiographical memory retrieval is emotionally loaded, the free retrieval, therefore, might activate emotional material in our participants. In other words, when asked to describe the memory that comes to mind first, as was the case in the free autobiographical condition, participants might have retrieved emotional memories. This interpretation can be supported by a previous study that reported that the majority of autobiographical memories cued by neutral words were emotional [51]. Because autobiographical memory typically triggers emotional material [31, 63], the lack of significant differences of pupil dilation between the free autobiographical memory retrieval compared with the positive and negative retrieval can be attributed to the assumption that all the three conditions might triggeremotional material.

In our view, similar pupil dilation for freely recalled, positive and negative memories may be attributed to the fact that our study, and that of Schaefer and Philippot [46] and Marci, Glick [64], included subjects without affective disorders. These subjects may succeed to regulate the emotional load of memories. It would be of interest therefore to replicate our procedures with a clinical population. For instance, because patients with depression have demonstrated systematic bias that favours negative memories [65, 66], it would be of interest to assess pupil dilation for negative memories in these patients.

## Venues for Future Research

As previously mentioned, one venue for future research would be to assess pupil dilation during autobiographical memory retrieval in pathological populations. The assessment of pupil dilation would be especially valuable in amnesia. For instance, patients with Alzheimer’s disease have severe difficulties to retrieve autobiographical memories [67, 68, 69] and the same thing can be said for patients with Korsakoff’s syndrome [70, 71, 72, 73, 74]. It would be of interest to investigate whether pupil dilation would be observed despite autobiographical memory retrieval in these patients.

Also, both Alzheimer’s disease and Korsakoff’s syndrome are characterized by false memories [75, 76, 77, 78, 79, 80, 81, 82, 83, 84, 85, 86]. It would be of interest to evaluate whether these memories would activate pupil dilations because research has demonstrated pupil dilation for false memories in normal populations. For instance, Montefinese, Ambrosini [87] reported higher pupil dilation for false alarms (i.e., items erroneously judged as old) than misses (i.e., items erroneously judged as new).

We would also like to emphasize that pupil dilation, as observed in our study, reflects the general physiological characteristics of this dilation. As mentioned in the introduction, pupil diameter typically varies from 1.5 to nine mm, and, in standard light conditions, pupil diameter is about 3 mm [3]. Also, the difference differences on pupil dilation between the autobiographical and control conditions mirrors that observed in research on pupil dilation and cognition; pupil typically dilates around 0.5 mm to cognitive stimulation [2].

## Conclusions

Pupil activity has been considered as a “summed index” of brain activity during cognitive processing. Cognitively relevant pupil activity typically occurs following inhibition of the parasympathetic nervous system as controlled by the locus coeruleus– norepinephrine system, which plays a key role in the regulation of cognition. By demonstrating how autobiographical memory can influence pupil activity, our study shows how pupillometry can be used as a measure of physiological responses to retrieval of self-related information.
